# Uveitis in the Pediatric Population and Therapeutic Management: A Current Literature Review

**DOI:** 10.3390/children11070769

**Published:** 2024-06-25

**Authors:** Monika Modrzejewska, Oliwia Zdanowska, Dawid Świstara, Piotr Połubiński

**Affiliations:** 1 2nd Department of Ophthalmology, Pomeranian Medical University in Szczecin, Powstańców Wielkopolskich 72, 70-111 Szczecin, Poland; 2 K. Marcinkowski University Hospital in Zielona Góra, 65-046 Zielona Góra, Poland; 3 Scientific Association of Students, 2nd Department of Ophthalmology, Pomeranian Medical University in Szczecin, Powstańców Wielkopolskich 72, 70-111 Szczecin, Poland

**Keywords:** uveitis in children, treatment of uveitis in the pediatric population, treatment of uveitis complications

## Abstract

Uveitis is an inflammatory disease that can lead to severe complications, including vision loss. The pediatric population is particularly at risk of developing complications, as uveitis in this age group often has idiopathic origins or is associated with systemic diseases that follow a severe course. This, coupled with unfavorable treatment outcomes, continues to be a challenge in pediatric ophthalmology. The cornerstone of uveitis treatment involves a therapeutic strategy that depends on the etiology, severity, and localization of the inflammation, as well as the patient’s response to treatment and the presence of ocular complications. Patients who do not receive timely treatment face a significantly increased risk of experiencing a severe disease course. Understanding potential therapeutic options and their side effects is crucial in managing children with uveitis. Equally important is the continuous monitoring of the child’s condition throughout the treatment process, due to the chronic and recurrent nature of uveitis in this demographic. The authors conducted a review of the current literature from 2018 to 2023 on the management and introduction of new therapeutic approaches for children with uveitis.

## 1. Introduction

Pediatric uveitis accounts for 5–10% of all uveitis cases [1]. In the pediatric population, the condition most frequently manifests as idiopathic anterior uveitis, representing about 29% of pediatric uveitis diagnoses, with juvenile idiopathic arthritis (JIA) being the second most common form, accounting for approximately 21% of cases. Uveitis in children may rarely have an infectious origin, estimated to constitute 6% of diagnoses in this age group [2]. The insidious onset and chronic nature of intraocular inflammation, coupled with the challenges of communication and conducting examinations in young children, make diagnosis and early treatment initiation difficult. Untreated inflammation can result in serious complications, such as cataracts, glaucoma, macular edema, and keratopathy [3]. The young body’s ongoing changes, psychomotor development, and potential for significant drug-related complications necessitate close monitoring of uveitis in children. Given the variety of systemic conditions that can underlie uveitis and the broad spectrum of potential side effects from therapeutic drugs affecting multiple organs, care for young patients requires a multidisciplinary approach.

The authors reviewed literature from 2018 to 2023, utilizing PubMed and Google Scholar to research the available therapeutic approaches for uveitis in the pediatric population. Owing to the limited number of studies involving this patient group, some of the included publications concern individuals beyond this age range.

## 2. Discussion

### 2.1. Treatment of Uveitis of Infectious Origin

#### 2.1.1. Toxoplasmosis

The standard treatment for toxoplasmosis relies on a triple-drug regimen, including pyrimethamine, sulfadiazine, and glucocorticosteroids (GCs) for 6 weeks [4]. The primary objective of antimicrobial therapy during the active phase of infection is to inhibit the proliferation of parasites. In chronic infections, antiparasitic medications may be ineffective against cysts [5]. Steroid eye drops are utilized in treating anterior segment uveitis, with their dosage adjusted based on the intensity of the inflammatory activity. Alongside topical steroids, mydriatics (pupil-dilating drugs) and hypotensive medications are prescribed. Mydriatics play a crucial role in preventing (or breaking) posterior synechiae and alleviating pain. In a randomized study by C. Slivier et al., in 2002, involving 124 patients with recurrent uveitis due to toxoplasmosis, 64 were administered 800 mg of sulfamethoxazole plus 160 mg of trimethoprim every third day for 20 months. The study found that reinfection occurred in 4 patients (6.6%) in the treated group and 15 patients (23.8%) in the control group [6]. Ten years after the study’s conclusion and treatment, a follow-up examination of the same patients revealed a recurrence of toxoplasmosis in 36–37% of participants in both groups, suggesting that the therapeutic effect diminishes once prophylaxis is halted [7]. Side effects of the medications used for toxoplasmosis treatment include leukopenia and thrombocytopenia. Blood tests should be conducted on a weekly basis throughout the treatment period, and folic acid levels must be closely monitored [4]. Sulfadiazine, a sulfonamide antimicrobial, may induce hypersensitivity reactions, including skin rashes.

#### 2.1.2. Lyme Disease

There are no standardized guidelines in the literature for treating uveitis in Lyme disease. Doxycycline, whether administered orally or intravenously, which has proven effective for neuroborreliosis, exhibits poor intraocular penetration. Due to this limitation, French guidelines for Lyme disease treatment advise against the use of doxycycline for intraocular infections [8]. Among antibiotics administered intravenously that achieve better intraocular penetration are meropenem, cephalosporins, ceftriaxone, cefazolin, and ceftazidime [9]. The literature also notes the intraocular efficacy of orally administered moxifloxacin and linezolid. Additionally, studies from 2019 and 2021 highlight the potential advantages of trimethoprim therapy for ocular Lyme disease and the investigation into disulfiram for the long-term management of recurrent Lyme disease [9,10]. Other therapeutic approaches may include the intravitreal injection of antibiotics such as ampicillin, oxacillin, cefazolin, vancomycin, ceftazidime, clindamycin, erythromycin, and moxifloxacin. This intravitreal antibiotic therapy may be augmented with intravenous drug administration to extend its effectiveness [11]. One of the more commonly described complications of antimicrobial therapy is the Łukasiewicz–Jarisch–Herxheimer Reaction, characterized by high fever, chills, increased sweating, hypotension, and cyanosis, usually developing within 24 h. To prevent this reaction before starting antimicrobial treatment, the administration of systemic or topical steroids is indicated [12]. The available literature does not contain a regimen of therapy for pediatric uveitis in the course of Lyme disease. Data from the literature relate mainly to the adult population or the general population without distinction by age.

#### 2.1.3. Herpes Simplex Virus

Antiviral medications and GCs are utilized in treating uveitis caused by the herpes simplex virus (HSV). Monotherapy with these drugs has been demonstrated to have comparable effectiveness. Studies have shown that acyclovir and valacyclovir possess similar efficacies, with oral acyclovir being more effective than its topical counterpart [13]. A randomized double-blind clinical trial in 1991 involving 86 patients with HSV infection revealed that a 7-day treatment with 800 mg of acyclovir administered five times daily had the same clinical outcomes as a 14-day treatment at the same dosage. Consequently, extending therapy with oral acyclovir offers little additional benefit [14]. Ocular side effects reported in the literature following systemic acyclovir use include conjunctivitis (54%), punctate keratitis (39%), dendritic epithelial keratitis (11%), corneal creeping ulcer, keratoconjunctivitis (13%), epithelial keratitis (1.9%), elevated intraocular pressure (13%), uveitis (16.7%), and eyelid and facial edema (5.6%). However, these studies pertain to the general population, with no detailed data specifically for the pediatric population available [15]. The most severe ocular complication of HSV infection in children, which can occur at any age, is acute retinal necrosis. This condition is marked by rapidly advancing necrotizing retinitis, predominantly in the periphery, accompanied by retinal and choroidal vasculitis and moderate to severe vitreous inflammation. Treatment involves the administration of antiviral medications both intravenously and intravitreally (in cases of severe vision-threatening retinitis or when there is involvement of the optic disc or macula), and systemic steroids are introduced after the commencement of antiviral therapy or once necrosis resolution is confirmed through physical examination [16].

#### 2.1.4. Cat Scratch Disease

Infection caused by Bartonella henselae, the etiological agent of cat scratch disease (CSD), typically resolves on its own in individuals without immunodeficiency. The literature lacks definitive guidelines for treating the ocular manifestations of this condition [17]. Systemic antibiotic therapy is advised for immunocompetent patients, those with severe systemic illness, or individuals facing severe visual loss threats, such as optic neuritis or retinitis pigmentosa.

The first-line antibiotics for treating CSD include doxycycline, tetracycline, and erythromycin, with ciprofloxacin, rifampicin, and trimethoprim-sulfamethoxazole serving as second-line treatments [18]. A multicenter cohort study by Habot-Wilner et al., conducted in 2018, examined the effectiveness of combined therapy with systemic antibiotics and corticosteroids in patients with moderate-to-severe ocular CSD. It confirmed that combination therapy significantly improved visual acuity compared to antibiotic therapy alone [19]. In 2015, Manousaridis et al. reported on a case of CSD-related macular edema treated with a single intravitreal injection of ranibizumab, underscoring the role of vascular proliferation in the pathogenesis of ocular CSD and suggesting the use of an anti-angiogenic drug as a viable treatment option for patients with CSD-related macular edema [20]. In 2023, Hong et al. identified surgery as an alternative treatment approach for complications arising from CSD. Diagnostic and therapeutic vitrectomy is advised for patients experiencing worsening vitreous opacity, rapid progression of retinal lesions, or retinal detachment [21]. Moreover, the effectiveness of combination therapy with doxycycline and steroids was demonstrated through the case of an 11-year-old boy with right-eye uveitis and bilateral nodular lesions on the fundus attributed to CSD [20].

#### 2.1.5. Tuberculosis

As of today, the treatment of ocular tuberculosis with antimycobacterial drugs remains controversial, with no definitive treatment regimen established [22]. Standard anti-tuberculosis therapy (ATT) with pulmonary tuberculosis involves a 2-month course of either four or three drugs: isoniazid, rifampicin, pyrazinamide, and/or ethambutol. Following this initial period, treatment continues with two drugs, isoniazid and rifampicin, for an additional four months. The aim is to halt the progression of tuberculosis in its initial phase and, in the second phase, to eradicate any remaining mycobacteria [23]. For other forms of tuberculosis, such as those involving the central nervous system, treatment duration may extend to 12 months [24]. In a study by Annamalai et al., patients with tuberculous uveitis who began ATT therapy early showed a positive response. A treatment duration of 9–12 months yielded favorable outcomes for both pulmonary and extrapulmonary TB forms. It was also noted that the risk of treatment failure and complications increased in cases with choroidal involvement and vitreous opacity [25]. Complications from ATT occur in about 7% of patients, including hepatotoxicity (6%) and skin rash (0.6%). Additional side effects, with a total incidence of about 0.4%, include gastrointestinal complaints, ocular toxicity (primarily affecting the optic nerve), and angioedema, among others. Isoniazid may cause neurological toxicity and hepatitis. Rifampicin can lead to gastrointestinal reactions, thrombocytopenic purpura, and, in rare cases, acute renal failure, dyspnea, shock, and hemolytic anemia. Pyrazinamide is known to cause arthralgia and hepatitis, while ethambutol’s side effects include optic neuritis outside the eye [26]. In 2020, as part of an international consensus initiative led by experts from the Collaborative Ocular Tuberculosis Study (COTS), in conjunction with the International Ocular Inflammation Society and the International Uveitis Study Group, recommendations for treating tuberculous uveitis were developed. It was acknowledged that current guidelines and the literature do not sufficiently guide physicians on initiating ATT for patients with tuberculous uveitis. Conversely, it has been established that physicians should consider initiating ATT when there is any immunological evidence of TB. However, there is a caution against the indiscriminate use of ATT for clinical presentations that do not definitively suggest a tuberculous origin, due to the rising concern over drug resistance and the potential for adverse effects from overuse in such scenarios. Beyond ATT, the use of steroids and immunosuppressive medications may be necessary to manage inflammatory responses. Oral steroid therapy is recommended to be commenced concurrently with or shortly after the initiation of ATT in patients with tuberculous serpiginous uveitis and tuberculous multifocal or unifocal uveitis. In instances of recurrent inflammation, systemic immunosuppressive therapy may be employed to facilitate the reduction in steroid dosage, thus minimizing corticosteroid use [27]. In a 2022 study, Bigdon et al. incorporated disease-modifying drugs such as methotrexate or azathioprine in the immunosuppressive regimen for patients with uveitis and latent tuberculosis, particularly when uveitis recurred during GC dose tapering or in cases of severe inflammation at the initial presentation. If a relapse occurred after 3 months of treatment, biologic therapy with adalimumab was also introduced [25].

#### 2.1.6. Candidiasis

The Royal College of Ophthalmologists’ Professional Standards Committee’s treatment guidelines for candidiasis are tailored to the infection site. For uveitis, amphotericin B is the recommended treatment. For deeper ocular structure infections, fluconazole is preferred due to amphotericin B’s limited ability to cross the blood–retinal barrier [28]. Immediate treatment initiation post-diagnosis is crucial; a study by Hautala et al. demonstrated a significant rise in ocular candidiasis development in patients whose treatment was delayed by at least 12 h compared to those who received earlier treatment [29]. Research has validated the effectiveness of vitrectomy in treating uveitis in the absence of candidemia [30]. Over time, Candida has developed resistance to various antifungal drug groups, leading to drug-resistant strains, largely attributed to antifungal overuse. Currently, multiple antifungal drug classes are available [31]. The introduction of new drugs is challenged by fungi’s eukaryotic cell structure and the pharmaceutical industry’s limited interest in developing new treatments [32].

### 2.2. Treatment of Non-Infectious Uveitis

The most common causes of non-infectious uveitis in the pediatric population are idiopathic and juvenile idiopathic arthritis-associated uveitis, pars planitis, Behcet disease, sarcoidosis, and Vogt–Koyanagi–Harada syndrome, but the prevalence of different types of uveitis varies depending on the nationality and geographical distribution of the studied group [1,3].

The primary objectives in treating non-infectious uveitis are to reduce intraocular inflammation, prevent recurrences, and avert ocular complications. The treatment necessitates a multidisciplinary and causal approach, primarily utilizing glucocorticoids, followed by disease-modifying drugs. In cases that are severe and resistant to standard treatments, biologic therapy is employed.

#### 2.2.1. Glucocorticoids

Glucocorticoids represent the most frequently used drug group in treating non-infectious uveitis. These medications can be administered topically, periocularly, intravitreally, or systemically, based on the inflammation’s severity, patient compliance, and existing complications. Their primary function is to inhibit the production of pro-inflammatory cytokines. For anterior segment uveitis, topical therapy includes 1% prednisone, 0.1% dexamethasone, and 0.05% difluprednate, a highly potent medication, though its use is restricted due to common side effects like increased intraocular pressure [2,33]. However, topical steroid administration is associated with a high risk of side effects such as cataracts, glaucoma, or increased intraocular pressure. The literature suggests that the frequency of the localized administration of steroids in drops has an impact on the occurrence of drug-induced cataracts; administration of three drops of the drug per day significantly increases the risk of occurrence of this complication in children [34]. Additionally, children under 6 years of age are more exposed to the risk of post-steroid increases in intraocular pressure. The same complications apply to the injected drugs; this way of administering drugs, especially to young children, may require the use of general anesthesia, which further creates additional complications [2]. Triamcinolone acetonide, administered through periocular or intravitreal injection, targets deeper sections of the uveal tract [35]. To mitigate the risks of repeated injections, implants with extended release have been utilized in managing chronic uveitis. Examples include Ozurdex, a vitreous implant delivering 0.7 mg of dexamethasone over approximately 6 months, and Retisert, releasing fluocinolone acetonide (0.59 mg) over an extended period (about 3 years) [36]. Implants are fully biodegradable and present fewer complications compared to intravenous drug delivery [35]. When symptoms are not adequately managed with topical treatments, systemic therapy via oral or intravenous medications should be considered. Prednisone is the most commonly prescribed GC for systemic use, with an initial oral dosage of ≤7.5 mg/day to minimize potential side effects. However, it may be necessary to increase the dosage to 60–80 mg/day to achieve the desired clinical outcomes, especially in the treatment of the acute phase of inflammation [37]. In the available literature, we can find the following scheme of systemic administration of steroids in case of severe, bilateral course of inflammation: therapy can be initiated intravenously using, for example, methylprednisolone in children at a dose not exceeding 30 mg/kg in continuous infusion; then, treatment should be continued orally with 1–2 mg/kg of prednisolone. However, regardless of the source, long-term steroid use is not recommended, especially in children [2]. Glucocorticoid therapy can lead to significant side effects, including localized effects in the eye such as cataracts, glaucoma, and retinal and choroidal emboli, as well as systemic effects like bone necrosis, psychosis, peptic ulcers, gastrointestinal and skin candidiasis, hirsutism, striae, impaired wound healing, weight gain, hypertension, hyperglycemia, secondary adrenal insufficiency [38], and stunted growth and sexual maturation [38]. It is recommended that the child’s development is monitored using growth charts with a particular focus on growth to age; growth retardation should constitute an alarm sign that may influence decisions on how to continue treatment, and in extreme cases, it may be an indication for consideration of growth hormone therapy [3].

In cases where there is no therapeutic response or if the disease progresses despite treatment with high doses of GCs, consideration should be given to initiating therapy with disease-modifying drugs, including methotrexate, mycophenolate mofetil, and cyclosporine A.

#### 2.2.2. Methotrexate

Methotrexate (MTX) is a disease-modifying immunosuppressive drug that is well-tolerated and effective in the pediatric population. It is utilized in treating conditions such as ankylosing spondylitis, psoriasis, and juvenile idiopathic arthritis. For uveitis associated with JIA, methotrexate is the first-line treatment, recommended at a dosage of 10–15 mg/m^2^/day [39]. It can be administered either orally or via subcutaneous injection. Greater efficacy of the drug is described when administered subcutaneously rather than orally. Retrospective studies also suggest that methotrexate therapy for JIA may offer protective benefits against the development of uveitis [40]. For severe uveitis in JIA, a combination of MTX and anti-TNF-α biologic treatment is advised [41]. During MTX therapy, the concurrent use of folic acid is necessary to mitigate side effects at a dose of 5 mg once a week during administration of the drug, as well as for 3 months after the end of treatment [2].

The most serious side effects of the drug may include toxic effects on bone marrow, the liver, and the gastrointestinal tract. Therefore, during MTX therapy, blood morphology and liver parameters should be monitored every 1–2 weeks after starting treatment, and then every 1–3 months as treatment continues. Children undergoing therapy are also more susceptible to infections [2,3,42]. In cases where MTX is contraindicated, other disease-modifying drugs are considered.

#### 2.2.3. Adalimumab

Adalimumab, a recombinant human anti-TNF-α antibody, is utilized in treating JIA and uveitis, among other conditions. In 2016, it was approved by the US FDA as the first anti-TNF-α drug for non-infectious uveitis [43]. Suitable for children as young as 2 years old, adalimumab is a human antibody, which minimizes the risk of allergic reactions and allows for subcutaneous administration [44]. In the United States, the FDA has authorized adalimumab for treating chronic non-infectious anterior segment uveitis in children over 2 years old, specifically in cases where uveitis does not adequately respond to standard treatments [3]. In Poland, adalimumab has been approved for use within a drug program for treating non-infectious chronic persistent or recurrent uveitis when treatment with GCs and disease-modifying drugs proves ineffective or leads to complications [45]. The drug is indicated for uveitis associated with autoimmune diseases exhibiting HLA-B27 gene expression (such as JIA), sarcoidosis, or birdshot retinochoroidopathy oraz w JIA-associated anterior uveitis [46]. In the treatment of uveitis associated with Behçet’s disease, adalimumab does not interact with anti-rheumatic medications commonly used for the condition [47]. The combination of adalimumab with conventional therapy in Behçet’s disease uveitis has demonstrated superior therapeutic effectiveness in cases of refractory uveitis compared to conventional treatment alone [48]. The VISUAL III study revealed that after 150 weeks of therapy, the dosage of GCs could be reduced from 9.4–17.1 mg/day to 1.5–3.9 mg/day, thereby decreasing the incidence of GC-related side effects [49]. A cohort study by Kouwenberg et al. found that adalimumab treatment in pediatric non-JIA uveitis led to the cessation of inflammation in 91% of the children studied [50]. The dosing regimens of adalimumab in pediatric patients reported in the literature differ slightly from publication to publication. Tuğal-Tutkun et al. in their review suggest subcutaneous administration of 20 mg every 15 days for children weighing less than 30 kg, and 40 mg every 5 days for children weighing more than 30 kg [2]. For patients who do not respond to the primary treatment regimen, more frequent weekly dosing is recommended. In JIA-associated anterior uveitis the proposed dosage regimen is to repeat every 14 days the administration of 20 mg of adalimumab in children weighing less than 30 kg. In children with a higher body weight the same regimen as in adults is suggested, i.e., 40 mg [51]. The most commonly reported side effects of adalimumab, as well as other TNF-α inhibitors, include sepsis due to bacterial, fungal, and viral infections, opportunistic infections, pneumonias, pyelonephritis, septic arthritis, and tuberculosis [52]. In children with the highest frequency, there are minor infections, respiratory and gastrointestinal disorders [53,54]. It is particularly important in children to monitor the patient’s condition during therapy and to regularly monitor blood count, liver function, trough drug levels and antibodies and before starting treatment conducting diagnostic tests in the direction of HIV/HepB/HepC [3].

#### 2.2.4. Other Medications

Infliximab, similar to adalimumab, is a biologic medication and a monoclonal antibody that targets and neutralizes TNF-α. It serves as an alternative treatment for uveitis in cases where there is an inadequate response to combination therapy with steroids and adalimumab [55]. A meta-analysis spanning from 2005 to 2020 showed that stabilization or improvement in visual acuity was achieved in 96% of patients within 12 months. Among the 82 patients studied, GCs were discontinued in 68 due to uveitis remission. The most common side effects reported were allergic reactions and infections [2]. Depending on the publication, there are some differences in dosage in children: from 5–6 mg/kg in intravenous infusions repeated every 6–12 weeks [3] or from 3 to 5 mg/kg every 4 to 8 weeks [2,56]. The more advanced the disease process, the longer the duration, the less satisfactory the response to treatment with lower doses with a lower frequency of administration. In children with severe, treatment-resistant course of uveitis, the frequency of administration may be increased (every 4 weeks) and the dose may be increased up to 10 mg/kg [57].

Etanercept is a recombinant protein that combines the p75 TNF receptor with the Fc fragment of IgG1. It functions by preventing TNF-α from binding to cells, thereby inhibiting further inflammation. Compared to anti-TNF-α monoclonal antibodies, etanercept demonstrates lower efficacy and offers weaker protection against the initial onset or recurrence of uveitis associated with JIA [58,59].

Tocilizumab, a monoclonal antibody that inhibits the interleukin-6 receptor, is a promising option for treating non-infectious uveitis, route of administration may be intravenous or subcutaneous [60]. The APTITUDE trial assessed tocilizumab’s effectiveness in patients with uveitis associated with JIA, particularly in those who did not respond to combined treatment with anti-TNF-α and MTX. The study reported a positive therapeutic outcome in three out of four patients with uveitis resistant to conventional therapies [61]. There are literature reports on the effectiveness of the drug in the treatment of cystoid macular edema. This medicinal product has not yet been registered in any of the indications in the pediatric population [58].

In severe cases of uveitis, biologic medications are employed, encompassing TNF-α inhibitors and antibodies targeting interleukins as well as B and T lymphocytes. Prior to initiating therapy, it is crucial to screen for hepatitis and tuberculosis [34].

For the treatment of uveitis in children with disease-modifying drugs or biological therapy, it is recommended that 2 years of remission of the disease be maintained before treatment intensity is reduced. Early withdrawal of medication risks recurrence of the disease [43,62].

### 2.3. Macular Edema—The Most Common Complication of Uveitis

Macular edema (ME) is the most prevalent complication of uveitis, representing about 30% of all cases of uveitis, irrespective of the cause or age group [63]. In the pediatric population, ME occurs in 15% of cases of non-infectious uveitis [64]. According to anatomical classification, ME is notably the most frequent complication in cases of intermediate uveitis or panuveitis, accounting for 40–60% and 53–64% of cases in these categories within the general population, respectively [65]. For mild, typically unilateral ME, treatment may be confined to topical therapies involving steroids and non-steroidal anti-inflammatory drugs (NSAIDs), often used in combination with steroids. The literature lists topical medications such as 0.05% difluprednate, 1% prednisolone, 0.1% nepafenac, 0.1% diclofenac [66], 0.09% bromfenac (noted for its synergistic action with vascular endothelial growth factors—VEGF) [67], and 0.5% indomethacin [61,68]. Topical therapy also includes various forms of injections, such as sub-Tenon, subconjunctival, orbital floor, transseptal, and inframammary, with similar therapeutic outcomes across all these methods [63]. Triamcinolone acetonide is frequently used, administered periocularly at 40 mg and intravitreally at 20 mg/0.1 mL [64], alongside methylprednisolone acetate, particularly in studies focusing on the adult population. Intravitreal injections of triamcinolone have proven more effective than its periocular administration [69]. Extended-release implants, such as Ozurdex, which contains 0.7 mg of dexamethasone, offer activity for up to 6 months and can be an option for treating ME, even after vitrectomy procedures [70]. Retisert is a non-biodegradable fluocynolone acetonide (FA) implant, surgically placed into the vitreous [71], along with other formulations like Yutiq, containing 0.18 mg FA, and Iluvien, with 0.19 mg FA. All three implants facilitate drug release for up to three years. Should these methods prove ineffective, non-biologic, immunomodulatory agents such as methotrexate, tacrolimus, sirolimus, azathioprine, cyclosporine, mycophenolate mofetil, cyclophosphamide, and type I interferons (including IFN α-2a, IFN α-2b, and IFN β-1a subtypes) may be considered. Literature reports suggest that interferon (IFN β-1a) has shown greater efficacy than MTX in treating ME resulting from uveitis, particularly in reducing macular thickness [72]. Methotrexate and sirolimus are available for intravitreal injection, with sirolimus showing a better safety profile in this form compared to subcutaneous administration, although it requires repeated injections [73]. For ME resistant to steroid therapy and conventional immunosuppressive medications, biologic therapy is employed, utilizing agents such as the following:

- TNF-α inhibitors feature monoclonal antibodies such as infliximab, administered intravenously, and adalimumab, given subcutaneously (presently the sole systemic non-steroidal medication approved by the FDA for treating intermediate and posterior uveitis in patients older than 2 years [72]). Golimumab, though available, has limited evidence of efficacy [61]. The fusion protein etanercept is not recommended for uveitis treatment and its use is generally discouraged [72].
- Anti-IL agents include tocilizumab, which targets interleukin-6 (IL-6). Its effectiveness has been documented in adult studies, particularly for those refractory to standard therapies, though discontinuing the drug has been linked to relapse [74]. Gevokizumab, an anti-IL-1β medication, was evaluated in a 2018 study by Tugal-Tutkun et al. for preventing uveitis complications in Behçet’s disease. The study highlighted gevokizumab’s anti-inflammatory benefits and its protective role against the development of ME. However, it did not show significant superiority over the control group [75].
- Anti-VEGF therapy, including bevacizumab, ranibizumab, aflibercept, and faricimab, is noted in the literature for providing only temporary relief from ME, necessitating multiple repeat injections. This poses a particular inconvenience for pediatric patients, who may require general anesthesia for each administration. A study conducted by Mitchell et al. in 2020 highlighted the efficacy and safety of ranibizumab in treating patients with diabetic ME [76]. This finding offers hope for future advancements in this area and suggests the potential for expanding the use of ranibizumab to include treatment for ME as a complication of uveitis.

Therapies currently under clinical trials include the following:

- Filgotinib, a Janus kinase 1 inhibitor, is being studied for its potential application in treating non-infectious uveitis, among other conditions, with a focus on its impact on the incidence of complications [61,77].
- Acthar gel, known for its effectiveness in treating sarcoidosis, is under investigation to assess its efficacy in treating uveitis associated with sarcoidosis and its complication in the form of ME [61,78].
- Ustekinumab, already used for psoriatic arthritis and Crohn’s disease—conditions that can be accompanied by uveitis—is being analyzed for its effects on ME [61,79].

These trials represent ongoing efforts to expand treatment options for uveitis and its complications, highlighting the potential for new therapeutic strategies.

There is a notable absence of a specific treatment protocol for ME resulting from uveitis in children, with most research focusing on adult populations. The most comprehensive study on pediatric patients to date, conducted by Eiger-Moscovich et al. in 2019, involved 25 children with ME secondary to uveitis, aged between 5 and 12 years [80]. The study concluded that the primary etiology, the specific phenotype, or the treatment regimen did not significantly influence the rate of ME resolution or the final visual acuity outcomes. ME was observed to resolve along with clinical symptoms within approximately 3 months of therapy, irrespective of the chosen treatment method. The treatments evaluated included topical GC injections or dexamethasone implants, systemic GCs, and immunomodulatory medications such as methotrexate, azathioprine, mycophenolate mofetil, as well as TNF-α inhibitors like infliximab and adalimumab. The findings highlight the effectiveness of treating ME associated with non-infectious uveitis in children and suggest that the choice of therapy should be tailored based on the individual assessments of treating physicians. This study underscores the critical need for further research into the efficacy and side effects of specific therapies within the pediatric demographic [80]. In 2021, Hong Nguyen et al. presented a study on 21 pediatric cases (mean age about 10.5 years) of ME in the course of uveitis [64]. The results mirrored those of the 2019 study by Eiger-Moscocich et al. [80] The treatment regimens varied, encompassing topical and systemic GC therapy, immunomodulatory therapy, and biologic therapy. After 12 months, remission was achieved in 69.2% of the cases. It was observed that GC injections led to a four-fold higher rate of ME cessation compared to other treatments. Additionally, the remission rate was higher for patients who had previously been treated with GC injections [64].

In the course of uveitis, complications such as macular edema, keratopathy, synechiae, cataracts, or glaucoma may develop. If complications do not respond to pharmacological treatment, surgical treatment may be required. These types of surgical procedures are fraught with high risks and require a precise and individualized approach. The prognosis of patients undergoing surgery is improved by strict control of inflammation, but there are no structured methods of perioperative treatment in the literature.

Immunosuppressive therapies used to treat uveitis have the potential to increase the risk of postoperative infections. However, many studies encourage non-interruption of immunosuppressive therapy during the perioperative period and have shown good results in this treatment [81,82]. Literature reports suggest the perioperative use of localized and/or systemic steroid therapy. In addition, during the pre- and postoperative period, it is recommended to that topical antibiotics are used or intraoperative intracameral or subconjunctival antibiotics are administered. It is recommended that a period of at least 3 months with inactive inflammation before the surgery is maintained [83]. For surgical treatment of uveitic glaucoma in children, the main surgical options are glaucoma drainage device, trabeculectomy, angle surgery, minimally invasive glaucoma surgeries, and cyclodestructive procedures. Kalogeropoulos, D. et al. describe in their publication the possibility of using anti-scarring agents, due to more intensive regeneration processes in children, in order to increase the chance of a successful postoperative effect [84]. The course of the operation as well as postoperative treatment in children are made difficult due to cooperation issues, especially in the case of young children; the whole therapeutic process requires a great deal of commitment from the caregivers. In the case of uvetic cataracts in children, it is possible to perform cataract surgery. Sijssens, K. in their study concluded that with good control of perioperative inflammation, the implantation of an intraocular lens in well-selected cases is not associated with any increased risk of developing ocular complications compared to aphakic patients [85]. Lensectomies-vitrectomy and phacoaspiration performed with posterior chamber intraocular lens can be performed in children’s surgeries in case of complications such as preoperative hypotony, vitreous opacity, or the presence of cyclitic membrane, in which case the preferred recommended procedure is pars plana vitrectomy with lensectomy [86].

## 3. Conclusions

Identifying the etiological agent of uveitis is crucial for selecting the appropriate treatment regimen, necessitating a multidisciplinary approach, an understanding of the clinical manifestations of systemic diseases associated with uveitis, and awareness of potential complications. In children, the predominant etiology is non-infectious, where the first-line treatments are GCs, followed by disease-modifying drugs for standard cases. For severe, refractory cases, biologic therapy, particularly adalimumab, is preferred [87]. It is essential for pediatric uveitis patients to be under close specialist surveillance until remission is achieved, with recommendations suggesting observations at least every 8–12 weeks in the early stages of the disease [1]. During follow-up visits, assessing visual acuity and measuring intraocular pressure are critical, with values above 21 mmHg and below 6 mmHg indicating potential latent activity or chronic damage from past inflammation. Evaluating potential complications, such as macular edema (noted in 15% of non-infectious uveitis cases in children), cataracts, keratopathy, or epiretinal membrane, is crucial, employing specialized imaging techniques for accurate diagnosis. Additionally, monitoring blood count, liver function tests including AspAT and ALT levels, urea nitrogen, and creatinine is vital to identify any organ damage stemming from treatment or as a manifestation of systemic disease [88]. For pediatric patients, closely observing the child’s condition, growth, and strict adherence to the prescribed therapy is paramount.
